# Artificial intelligence in dentistry and dental biomaterials

**DOI:** 10.3389/fdmed.2024.1525505

**Published:** 2024-12-23

**Authors:** Dinesh Rokaya, Ahmad Al Jaghsi, Rohan Jagtap, Viritpon Srimaneepong

**Affiliations:** ^1^ Clinical Sciences Department, College of Dentistry, Ajman University, Ajman, United Arab Emirates; ^2^ Center of Medical and Bio-Allied Health Sciences Research, Ajman University, Ajman, United Arab Emirates; ^3^ Department of Prosthodontics, Gerodontology, and Dental Materials, Greifswald University Medicine, Greifswald, Germany; ^4^ Division of Oral and Maxillofacial Radiology, Department of Care Planning and Restorative Sciences, University of Mississippi Medical Center (UMMC) School of Dentistry, Jackson, MS, United States; ^5^ Department of Prosthodontics, Faculty of Dentistry, Chulalongkorn University, Bangkok, Thailand

**Keywords:** artificial intelligence, machine learning, deep learning, neural networks, medicine, dentistry, dental medicine, dental biomaterials

## Abstract

Artificial intelligence (AI) technology is being used in various fields and its use is increasingly expanding in dentistry. The key aspects of AI include machine learning (ML), deep learning (DL), and neural networks (NNs). The aim of this review is to present an overview of AI, its various aspects, and its application in biomedicine, dentistry, and dental biomaterials focusing on restorative dentistry and prosthodontics. AI-based systems can be a complementary tool in diagnosis and treatment planning, result prediction, and patient-centered care. AI software can be used to detect restorations, prosthetic crowns, periodontal bone loss, and root canal segmentation from the periapical radiographs. The integration of AI, digital imaging, and 3D printing can provide more precise, durable, and patient-oriented outcomes. AI can be also used for the automatic segmentation of panoramic radiographs showing normal anatomy of the oral and maxillofacial area. Recent advancement in AI in medical and dental sciences includes multimodal deep learning fusion, speech data detection, and neuromorphic computing. Hence, AI has helped dentists in diagnosis, planning, and aid in providing high-quality dental treatments in less time.

## 1 Introduction

**Figure 1:**
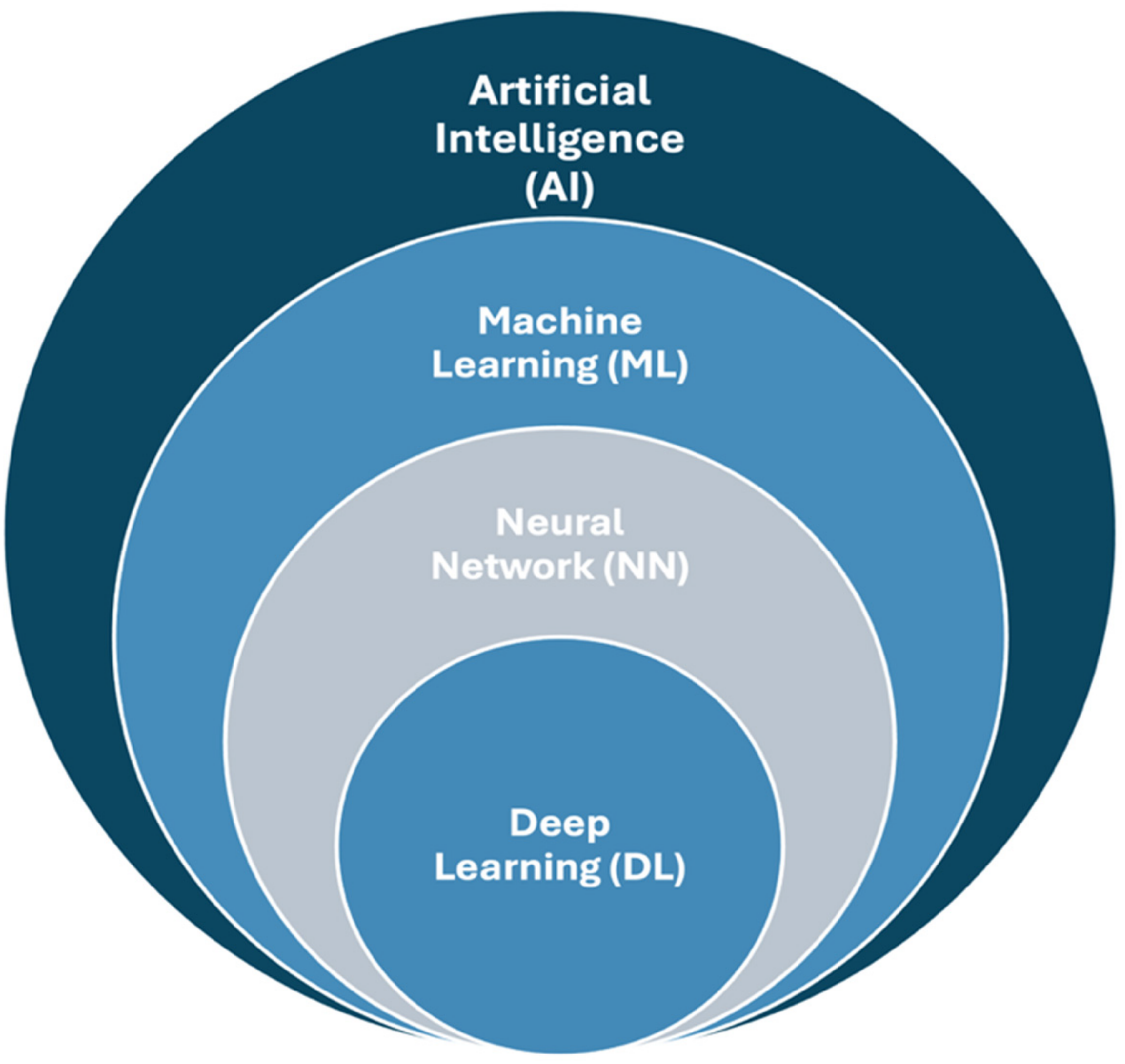
Key aspects of artificial intelligence; machine learning, neural network, and deep learning.

Artificial intelligence (AI) refers to the ability of machines to exhibit a form of intelligence (1). AI is described as “a branch of science and engineering concerned with the computational understanding of what is often referred to as intelligent behavior and the development of artifacts that display such behavior” (2). At present, AI has brought a new paradigm that affects various disciplines, including science and technology, and affects everyday life (3). AI uses machines to mimic human intellectual behavior and cognitive skills like problem-solving (1, 4). The key aspects of AI include machine learning (ML), neural networks (NNs), and deep learning (DL), as illustrated in Figure 1 (1). The wide applications of these materials include information, construction, biomedicine, and biomaterials (3). AI has made it possible to analyze large amounts of data (big data) in real time and provides forecasts that can support the clinician's decisions (5).

ML is part of AI, and it depends on algorithms that can predict outcomes from datasets. It is based on algorithms trained for decision-making that robotically learn and recognize patterns from data. ML facilitates machine learning from data, and it can resolve issues/problems without people's involvement (1). DL is a constituent of ML that involves algorithms that are inspired by the structure and function of the human brain's NNs (6). DL uses deep NNs, which are composed of multiple layers of interconnected nodes, and DL constructs NNs to identify patterns to improve feature detection (1, 6). DL uses convolutional neural networks (CNNs), which is an automated feature discovery from raw data, resulting in better generalization and real-time decision-making. Hence, DL is increasingly important in medical and dental research, particularly in areas such as radiological image classification and segmentation, brain mapping with fMRI data, and diagnostic prognostication using various data types. The goals of AI research are reasoning, knowledge, planning, learning, natural language processing, perception, and moving and manipulating objects (7). Such technologies require good medical image processing of digital data, effective interpreting of diagnostic images, and applying mathematical operations for calculation and interpretation (8).

AI has led to wide applications in the medical sciences and education (9–11). The use of digital dentistry and dental computer-aided design (CAD) and computer-aided manufacturing (CAM) technologies are being used in dental education in dental education curriculum (12). Nassani et al. (13) assessed the dental students' perception of digital technologies and CAD/CAM technologies integrating the dental students in scanning, designing, and manufacturing CAD provisional fixed dental restorations. They concluded that the presence of digital technology in practice and in educational academic environments significantly improved students' interest and perception of their knowledge and skills.

AI has been used in medical science. AI in imaging is one of the most developed areas for detection, classification, and splitting tasks in computer vision (14). AI is extensively utilized in medicine, where it serves a crucial function across various domains, including diagnosis and treatment planning, clinical care, laboratory processes, virtual assistant, treatment prognosis, educational training, administrative tasks, and electronic data record (EDR) (15). Recent advancements in AI in medical sciences include multimodal deep learning fusion (MDLF), speech data detection, and neuromorphic computing. At present, MDLF techniques are used in disease detection and diagnosis (16). The MDLF techniques can enhance the capabilities of machine learning models which can result in improved accuracy (17). These techniques provide adequate complementary information from multi-modal medical images and aid in disease diagnosis.

There is an increasing tendency of aging society with more elderly populations globally with more neurological and psychiatric disorders. Hence, there is a challenge to provide adequate care for many affected individuals, their families, and caregivers. AI has helped to address these problems to some extent. The interaction of neuromorphic computing and neuroscience results in understanding the human brain's complexities and addressing neurological challenges in elderly people. The body images are fused by using Siamese convolutional neural network structure and the entropy of the images (16). Therefore, the DL models can emphasize discerning complex patterns and offer advantages over conventional machine learning approaches. Recently, speech data have also been considered valuable clinical data for the detection of various diseases (18). AI-based techniques can help to categorize the data based on underlying algorithms. Such data helps to provide information on the association with the progressive degeneration of brain cells and successive impacts on cognition, memory, and language abilities. Various neurological diseases can be prevented, which can ultimately improve oral health. Finally, the development of neuromorphic computing has led to a transformative framework for modeling neurological disorders in drug development and therapeutic interventions (6, 19).

**Figure 2:**
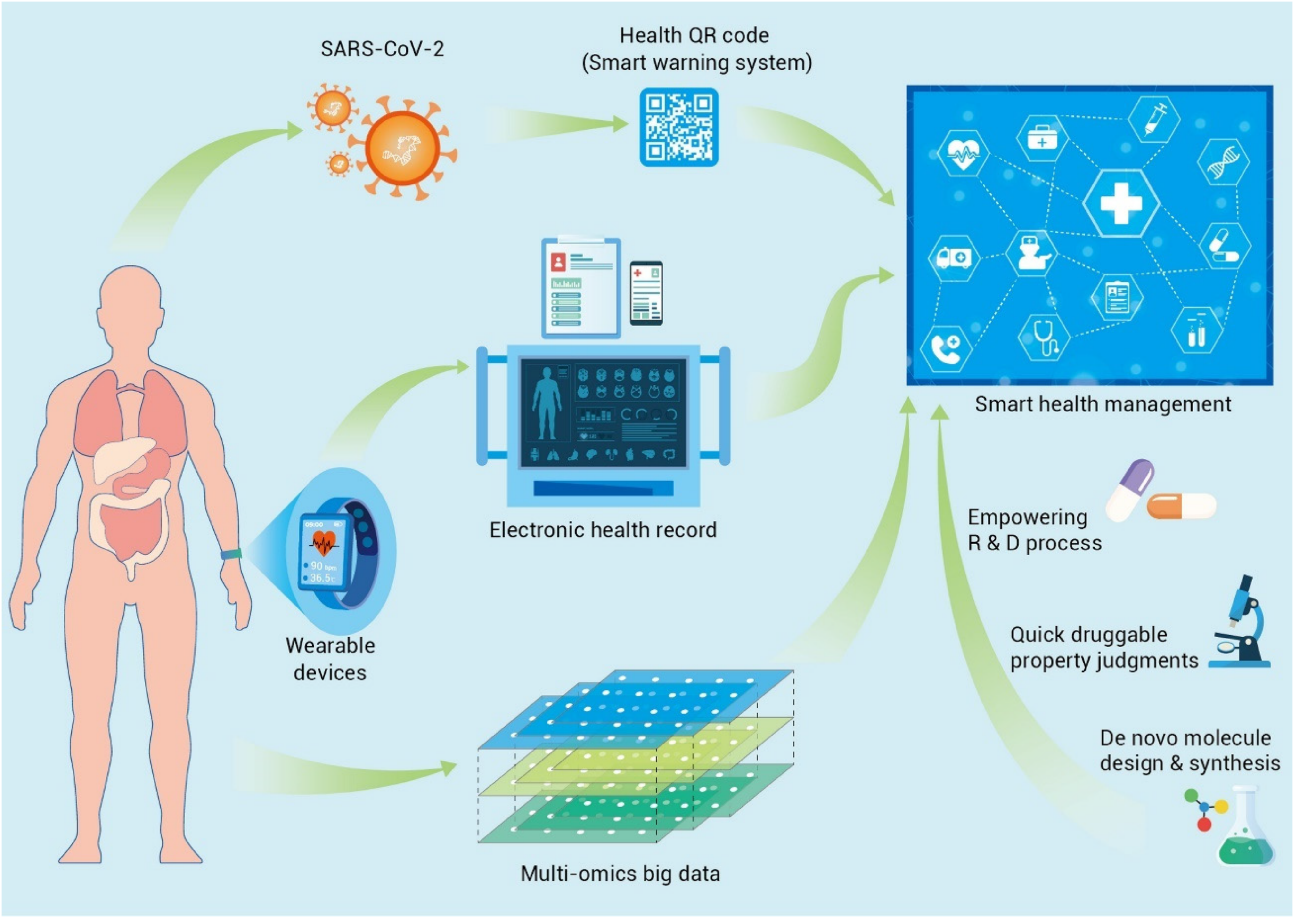
Applications of AI in medicine and dentistry. Adapted with permission from (3).

With the rising need for health care services, the needs of the hospital are evolving from traditional service-based to internet and smart hospitals (Figure 2) (3). AI can be implemented in public health services in various ways. One good example is the digital health Quick Response (QR) code, which uses a color-coding system to detect COVID-19 and show the person's health conditions using mobile data (20). This QR code was adopted by various countries to prevent and control pandemic diseases (especially COVID-19) worldwide.

## 2 Materials and methods

The aim of this review is to present an overview of AI, its various aspects, and its application in biomedicine, dentistry, and dental biomaterials focusing on restorative dentistry and prosthodontics. Articles on AI and its applications in medicine, dentistry and biomaterials were searched on PubMed, Google Scholar, Scopus, and ScienceDirect. Additional sources were also searched for additional articles and relevant articles were included in this review.

## 3 AI in dentistry

**Figure 3:**
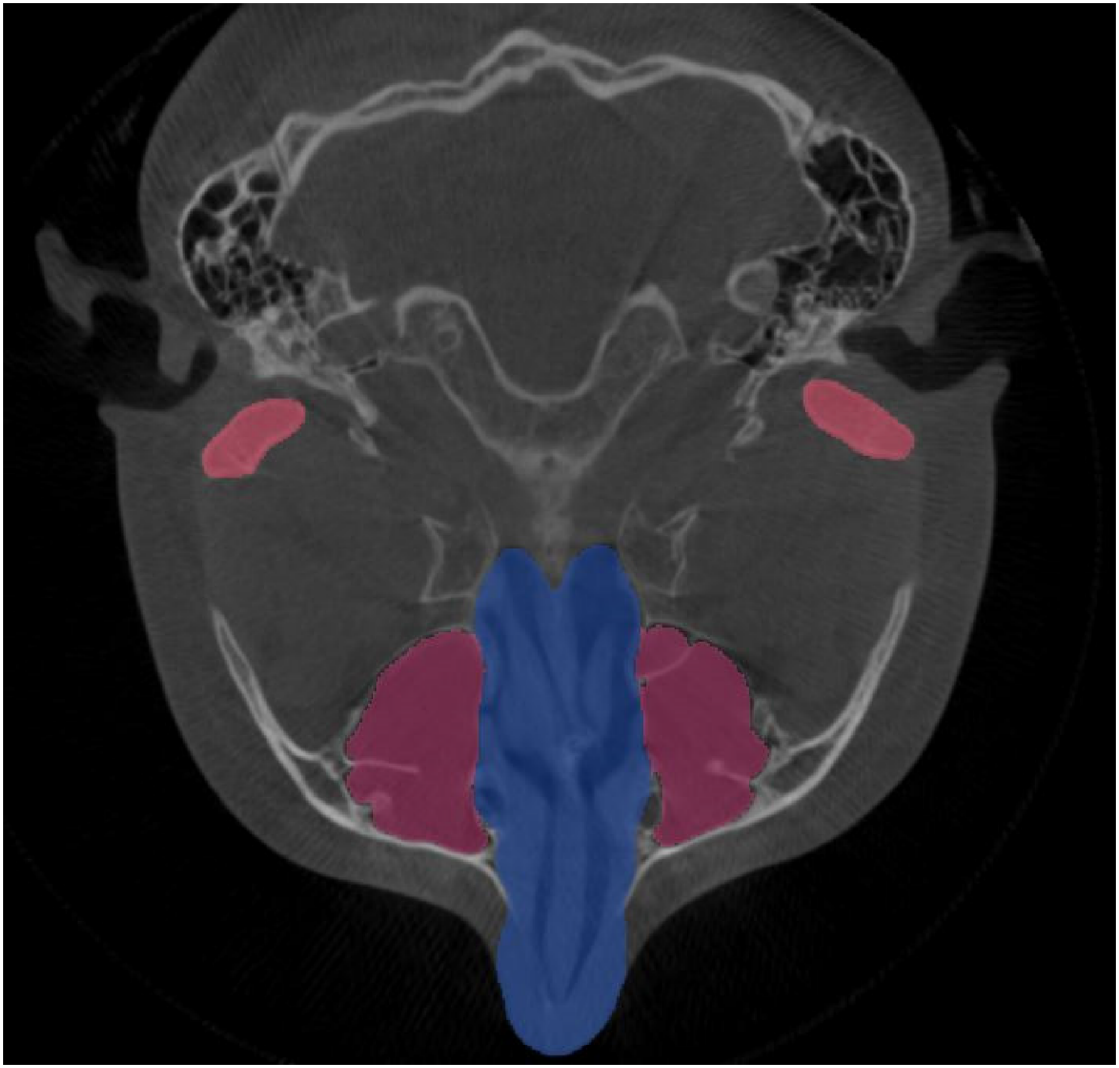
AI detection of various anatomical landmarks in the axial view of CBCT; the maxillary sinus, nasal cavity, and condyles.

In dentistry, AI is used in various specialties, i.e., maxillofacial radiology, orthodontics, prosthodontics, dental implantology, etc. (14, 21, 22). Modern dentistry is progressing into AI-assisted data-driven and robot-assisted. Various AI technologies have been applied such as robot dental assistants in oral surgery, tooth arrangement, computer-assisted orthodontics, and material testing (23). Diagnosis of diseases mainly relies on radiologists' subjective assessment and it varies according to clinical experience (8). AI models can be used to diagnose dental caries, root fractures, maxillofacial cysts, salivary gland diseases, maxillary sinusitis, osteoporosis, cancerous lesions, lymph node metastasis, and alveolar bone loss (1, 24). AI computer algorithms have also been applied for computer-assisted diagnosis and treatment using computed tomography (CT) and electrocardiography (ECG) repeatedly without fatigue (3, 22). The AI program anticipates pathology or prognosis by prioritizing risk factors (25). The first AI-based medical product permitted by the Food and Drug Administration (FDA) is IDx-DR, which uses an AI-based model based on patient images to predict diabetic retinopathy (26). Furthermore, an AI-based medical smartphone application called SkinVision can precisely identify melanomas (27). AI can be used to detect various anatomical landmarks, such as the maxillary sinus, nasal cavity, and condyles, as shown in Figure 3.

**Figure 4:**
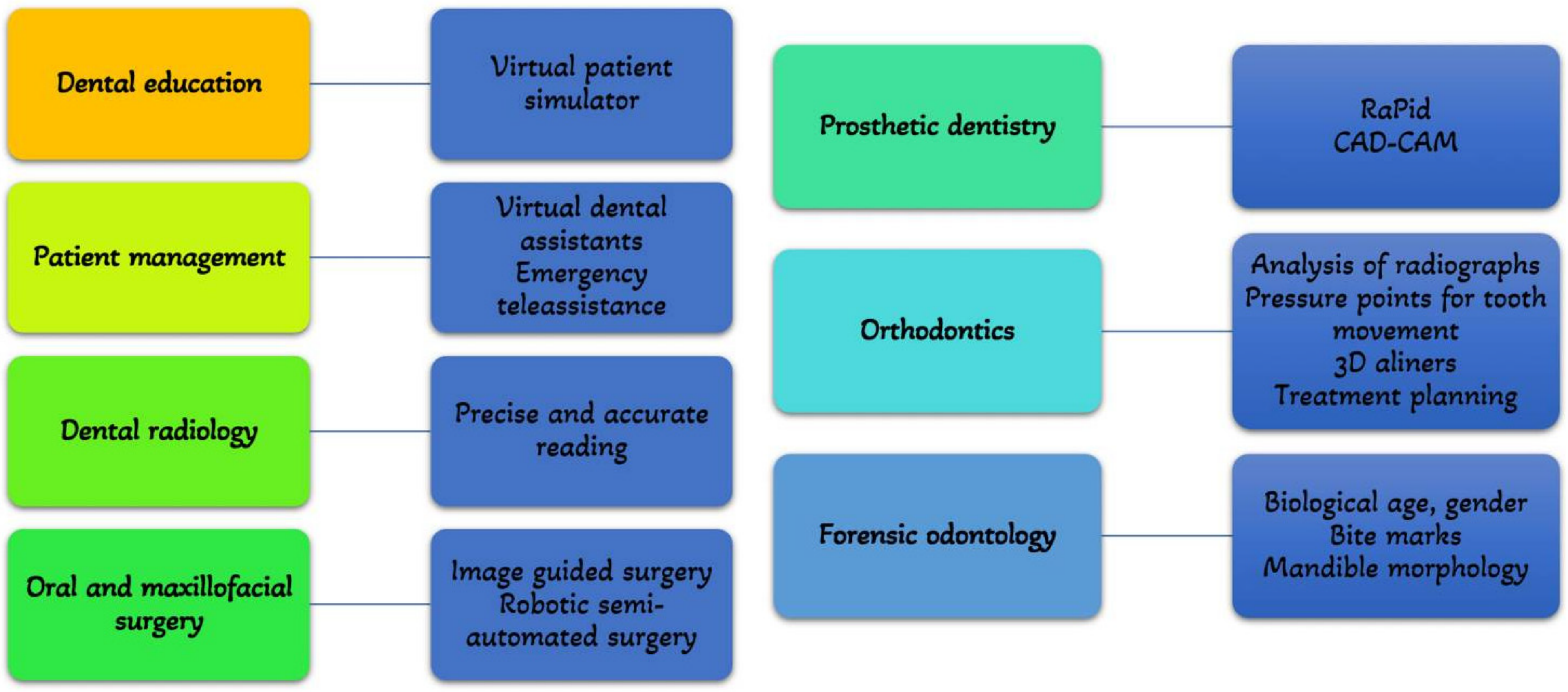
Applications of AI in dentistry. Adapted with permission from (25).

In orthodontics, AI models can be used to detect the need for orthodontic treatments, predict orthodontic extractions, and perform cephalometric analysis. In endodontics, AI models can be used to locate the apical foramen, assess root morphologies, predict retreatment, predict periapical pathologies, and detect root fractures (24). The different applications of AI in dentistry are illustrated in Figure 4 (25). AI is also useful in biological age and sex identification (25, 28).

**Figure 5:**
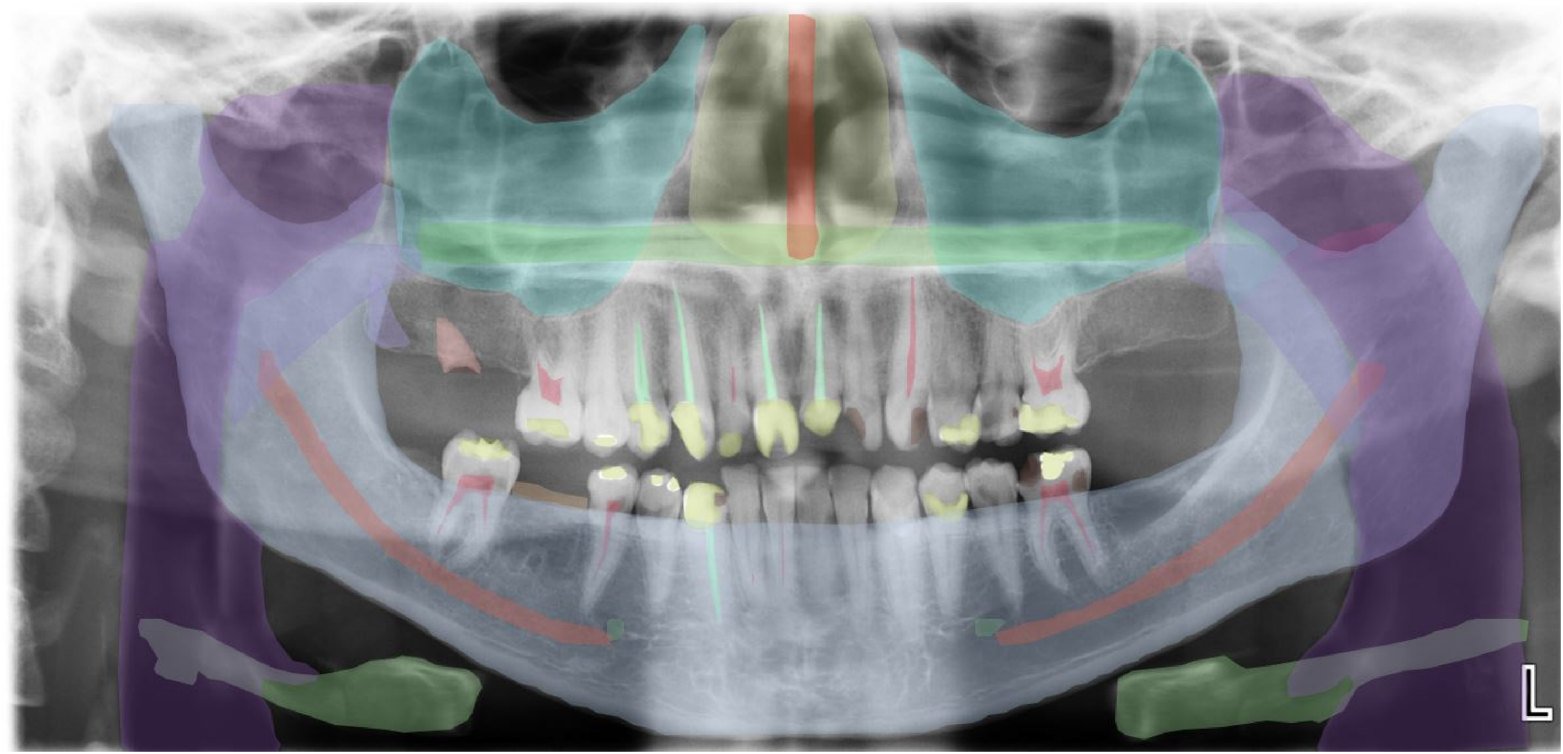
Automatic segmentation of a panoramic radiograph showing normal anatomy of oral and maxillofacial area.

**Figure 6:**
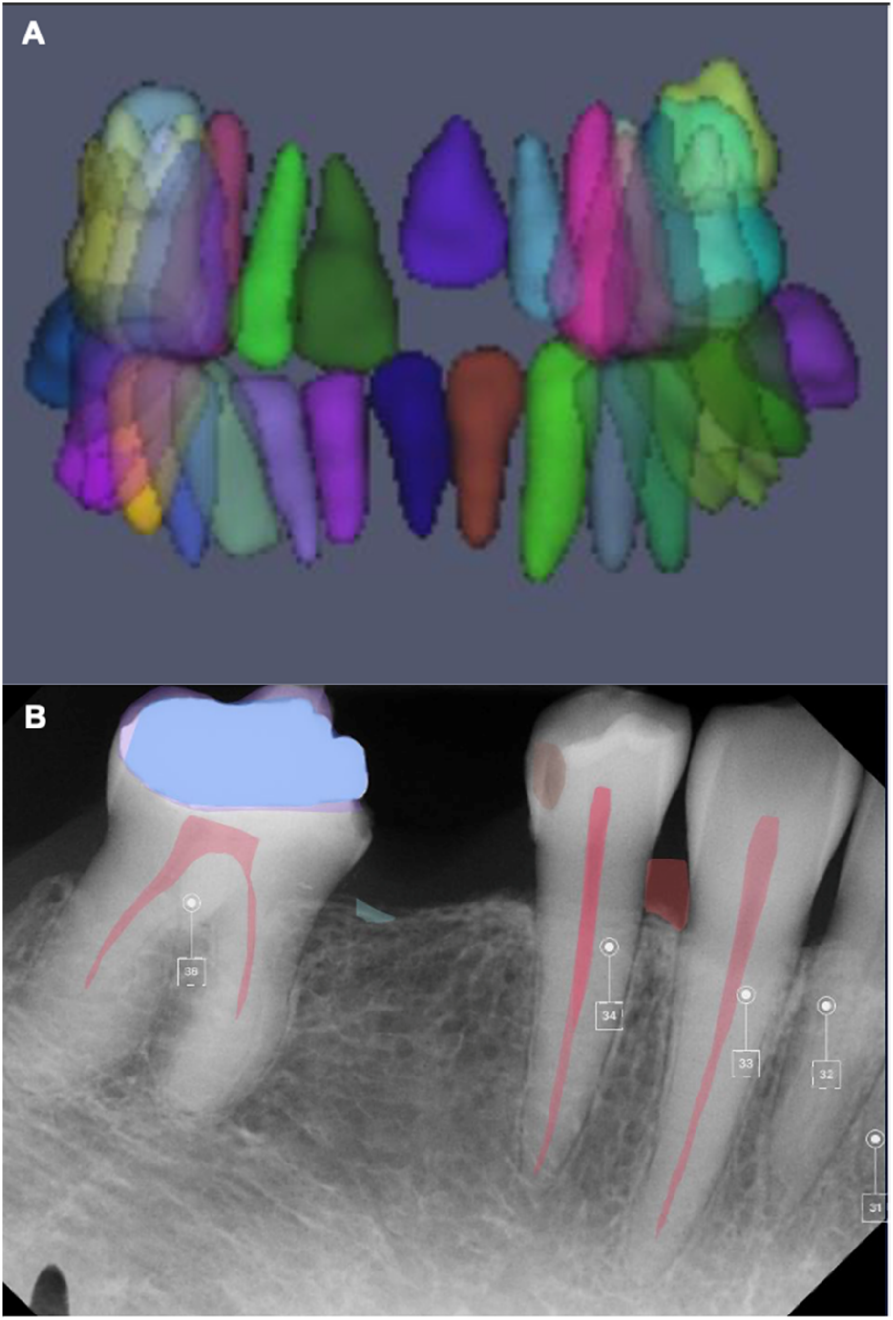
AI for tooth presentation in 3D view by an AI system **(A)** and automatic crown and caries detection on the periapical radiograph **(B)** (courtesy—cranioCatch AI software).

Furthermore, AI can be used to evaluate occlusal contacts and predict mandibular morphology (1). AI can also be used for the automatic segmentation of panoramic radiographs showing normal anatomy of the oral and maxillofacial area (Figure 5). In addition, AI is also used for tooth presentation in 3D view by AI systems and automatic crown and caries detection on periapical radiographs (Figure 6). Hence, dentists are increasingly relying on computer applications and artificial intelligence models for clinical decision-making (29, 30). This approach not only supports clinical decisions but also increases the precision, efficiency, and accuracy of treatment planning and oral rehabilitation (31).

### 3.1 AI in restorative dentistry and prosthodontics

**Figure 7:**
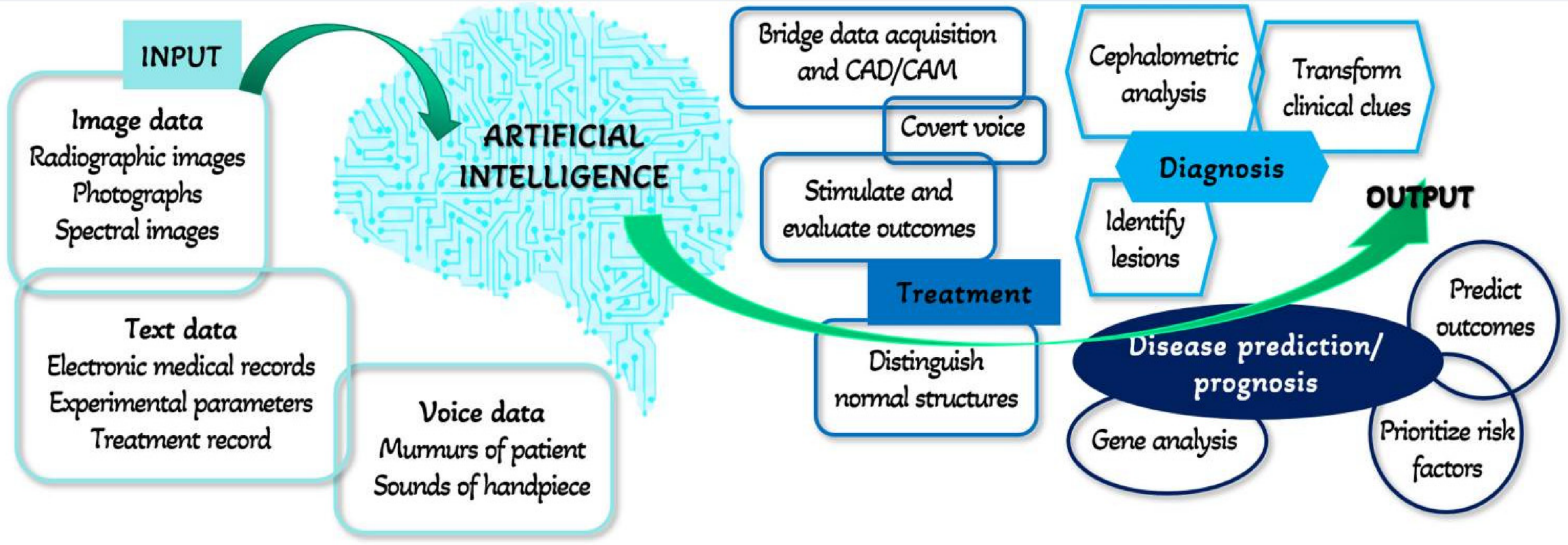
Hierarchy of AI in dentistry. Adapted with permission from (25).

Digital technologies and AI-based applications have streamlined dental care, simplified laborious routine tasks, increased health at lower cost, and enabled personalized and predictive dentistry (32, 33). AI functions like machines and follows a basic hierarchy; input, processing, and output, as shown in Figure 7 (25). In dentistry, data (experimental parameters and medical records) or pictures (photos and radiographic images). The results can be used for disease prediction, diagnosis, treatment, or prognosis. AI can predict treatment from the input by distinguishing normal structures, stimulating, and evaluating the outcomes (31, 34).

**Figure 8:**
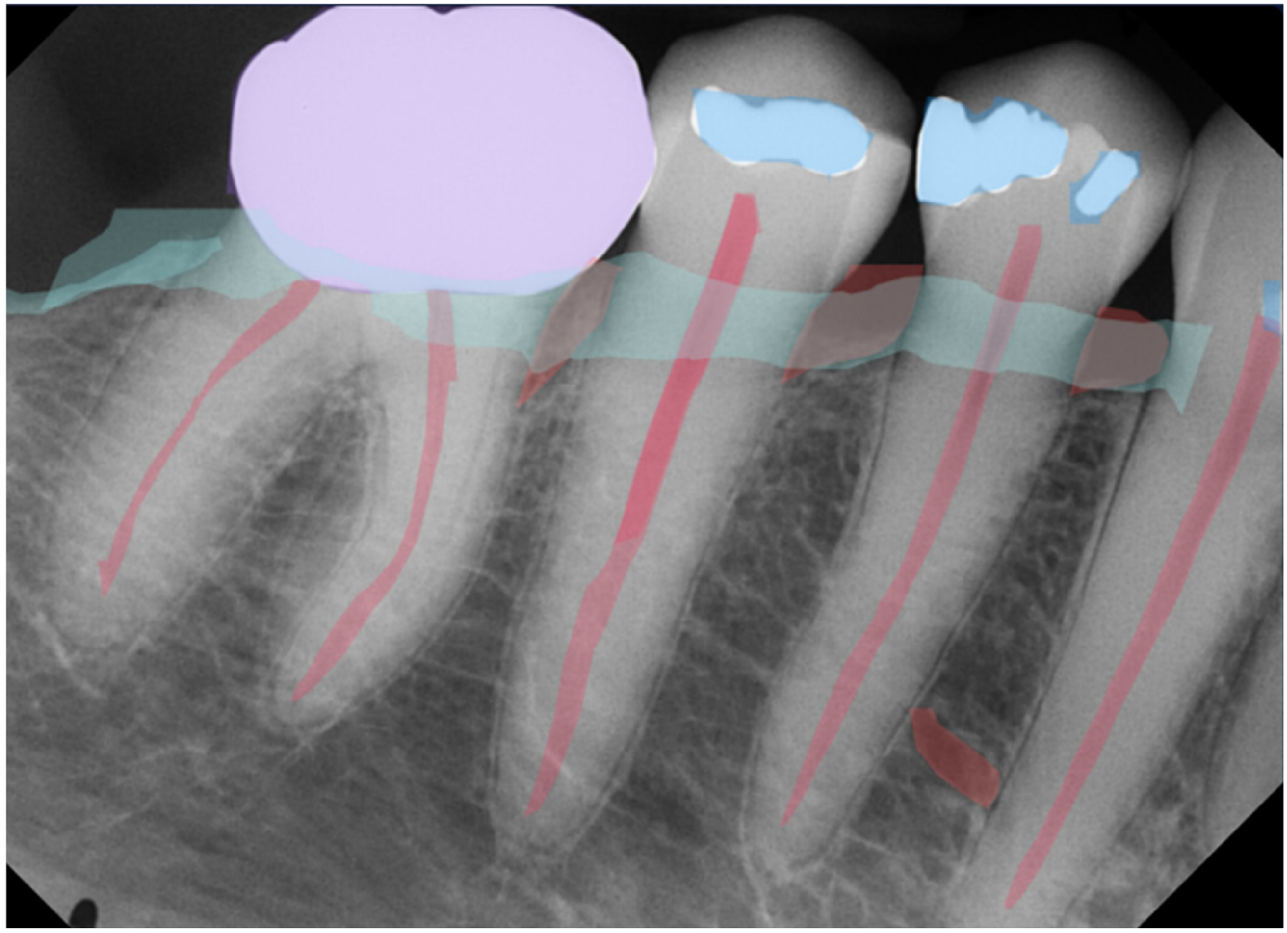
Restoration, crown, periodontal bone loss, and root canal segmentation as observed in periapical radiographs. (Courtesy—CranioCatch AI software).

**Figure 9:**
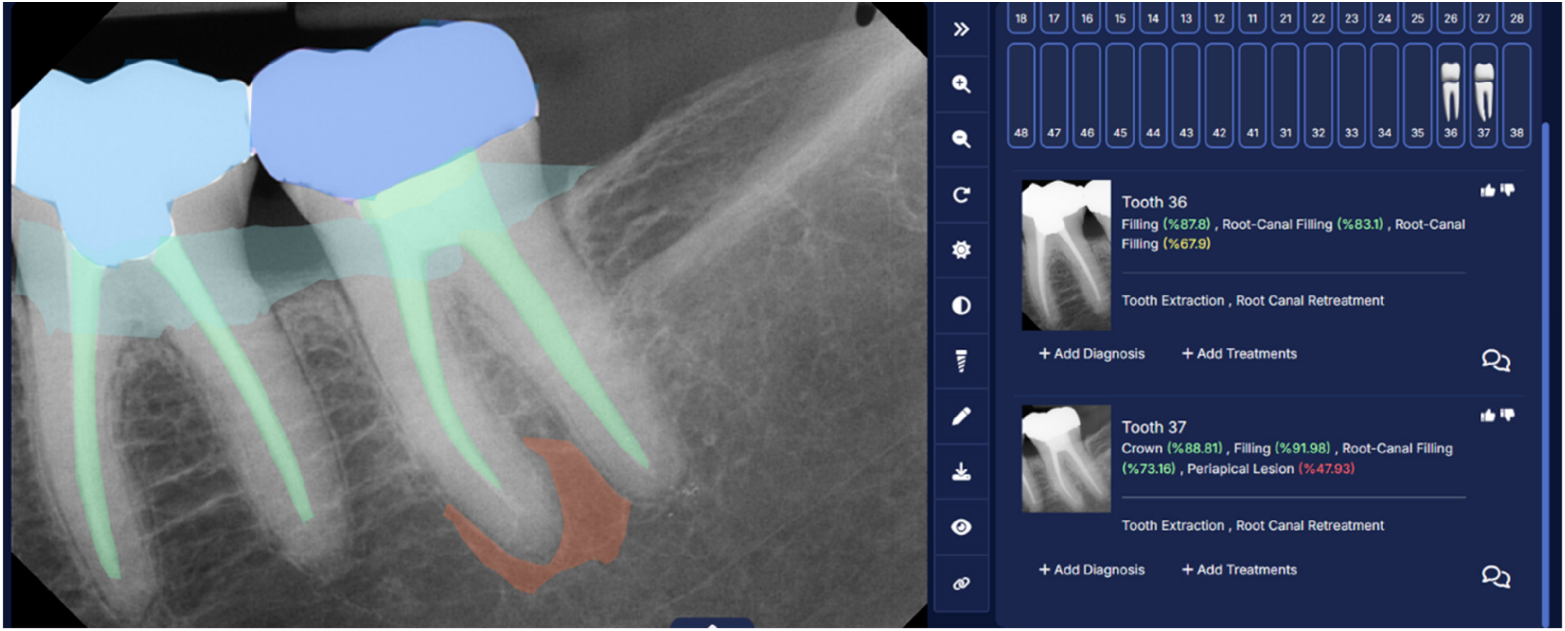
Identification and segmentation of a prosthetic crown and periapical radiolucency on a periapical radiograph. (Courtesy—CranioCatch AI software).

AI software can be used to detect restorations, prosthetic crowns, periodontal bone loss, and root canal segmentation from the periapical radiographs as shown in Figure 8. In addition, AI software can be used in the identification and segmentation of a prosthetic crown and periapical radiolucency on a periapical radiograph (Figure 9).

AI technology helps examine a patient's oral structure and customize treatment based on the patient's condition (35). AI can also recommend the most suitable biomaterials, designs, and technologies for prosthesis fabrication (36). Such customized prostheses have the advantages of good fit, comfort, and function (37). Furthermore, the incorporation of AI into 3D printing permits rapid fabrication of dental prostheses rapidly (36). Zirconia prostheses can be produced in-office on the same day using a speed sintering technique and delivered to the patient, providing rapid and cost-effective solutions for their patients (38–41). These use intraoral scanners and computer-aided design/computer-aided manufacturing software programs for prosthesis fabrication (40).

Furthermore, the computer-based prosthesis design uses the knowledge of bioengineering biomechanics and expert systems. A design-assisted computer application for removable partial dentures (RPD), RaPid, links knowledge-based systems, databases, and CAD systems (42). The patients' data are interpreted as transactions set on a database of design components. This application can be used in RPD and it has combined various parameters such as ethnicity, anthropological calculations, face proportions, and patient preferences for optimal esthetics. RaPiD uses design rules; that are implemented instantly when the design components are generated which describe characteristics such as the size and orientation of rests (the component that transfers force during mastication) and the connection between clasp arms and rests (retaining and reciprocating parts). The RPD design applications often require the manipulation of various component types, constraints, geometrical properties, and related computational methods. This software is useful in designing RPD.

**Figure 10:**
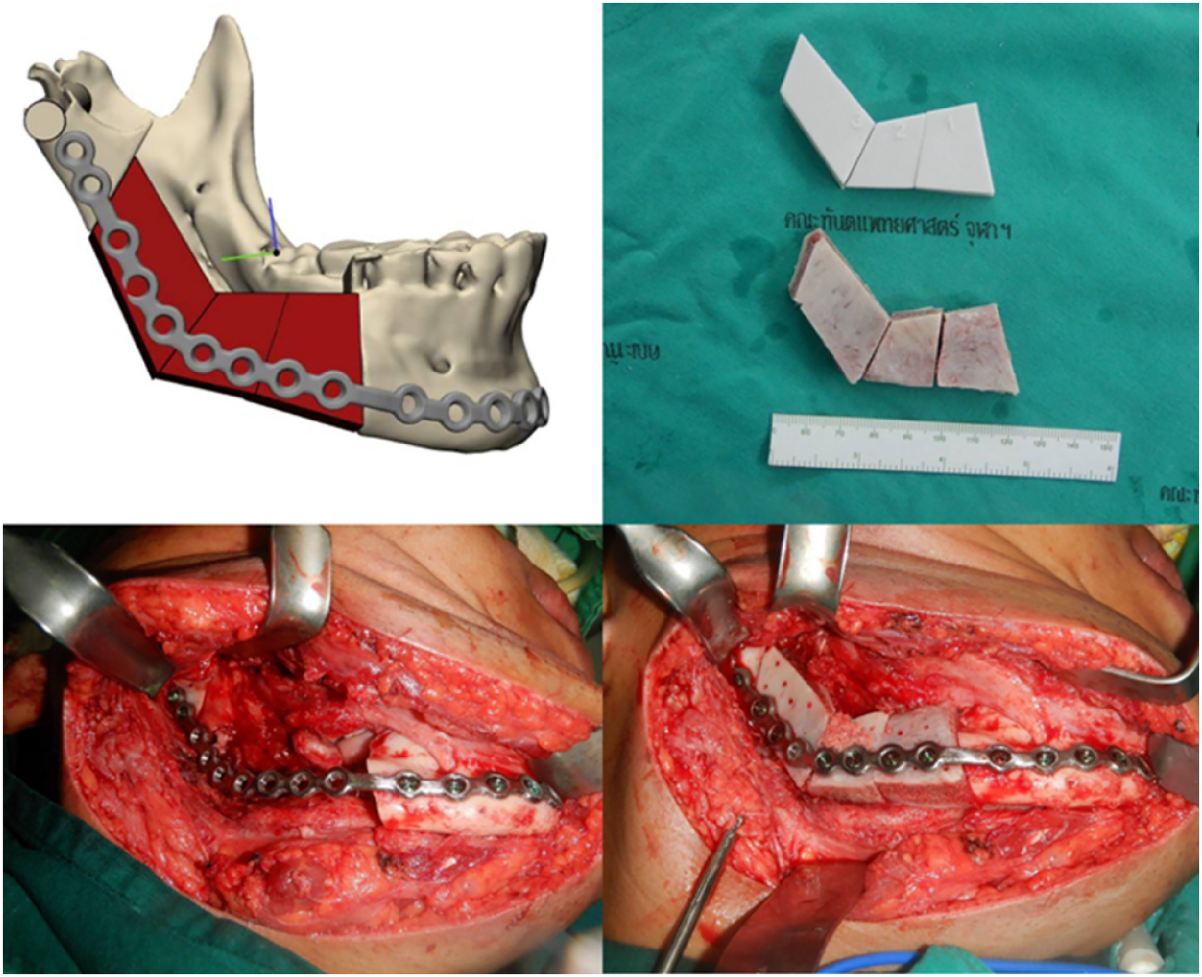
AI for creating a model of bone graft from the iliac bone for reconstructing mandibular defect (courtesy of Dr. Wichuda Kongsong).

Finally, with developments in NN, dental laboratories are using AI to create advanced dental restorations with high standards of fit, function, and esthetics (25, 43). AI is also being used for maxillofacial prostheses. With AI, a bone graft model can be created for surgical planning, and bone grafts can subsequently be created from the iliac crest. These bone grafts with reconstruction plates are used to reconstruct the mandibular defects (Figure 10).

### 3.2 AI in dental implant prosthodontics

AI has been used in dental implant prosthodontics. In dental implantology, AI is used in diagnostics, treatment planning, and patient outcomes (44). In addition, AI can enhance the precision of treatment planning, enable differentiation of implant brands through ML, help in designing the implants via finite element analysis, and predict treatment outcomes (44, 45). In addition, AI is an important tool for detecting anatomical landmarks that are important for implant placement. AI helps detection of the mandibular canal using a deep convolutional neural network model (46–48). This factor plays an important role in implant planning and helps prevent per- and postoperative neurovascular complications. Morgan et al. (49) used a convolutional NN for automatic segmentation of the maxillary sinus, which allowed for the exact reproduction of 3D models for planning.

For prosthetically driven implants, precise three-dimensional (3D) placement is necessary (44). AI helps in treatment planning for dental implants by helping in decision-making. Mangano et al. (50) combined AI and augmented reality for guided implant planning in a partially edentulous patient, and their protocol was time-saving and efficient. AI can be also used to predict the primary stability of dental implants (accuracy of 93.7%) based on drilling protocols during implant surgery, which is useful for young clinicians (51). Furthermore, AI can also help identify dental implants through the convolutional neural network of deep learning by forming an identification algorithm to detect spatial features such as shape, texture, and edges (52, 53).

**Figure 11:**
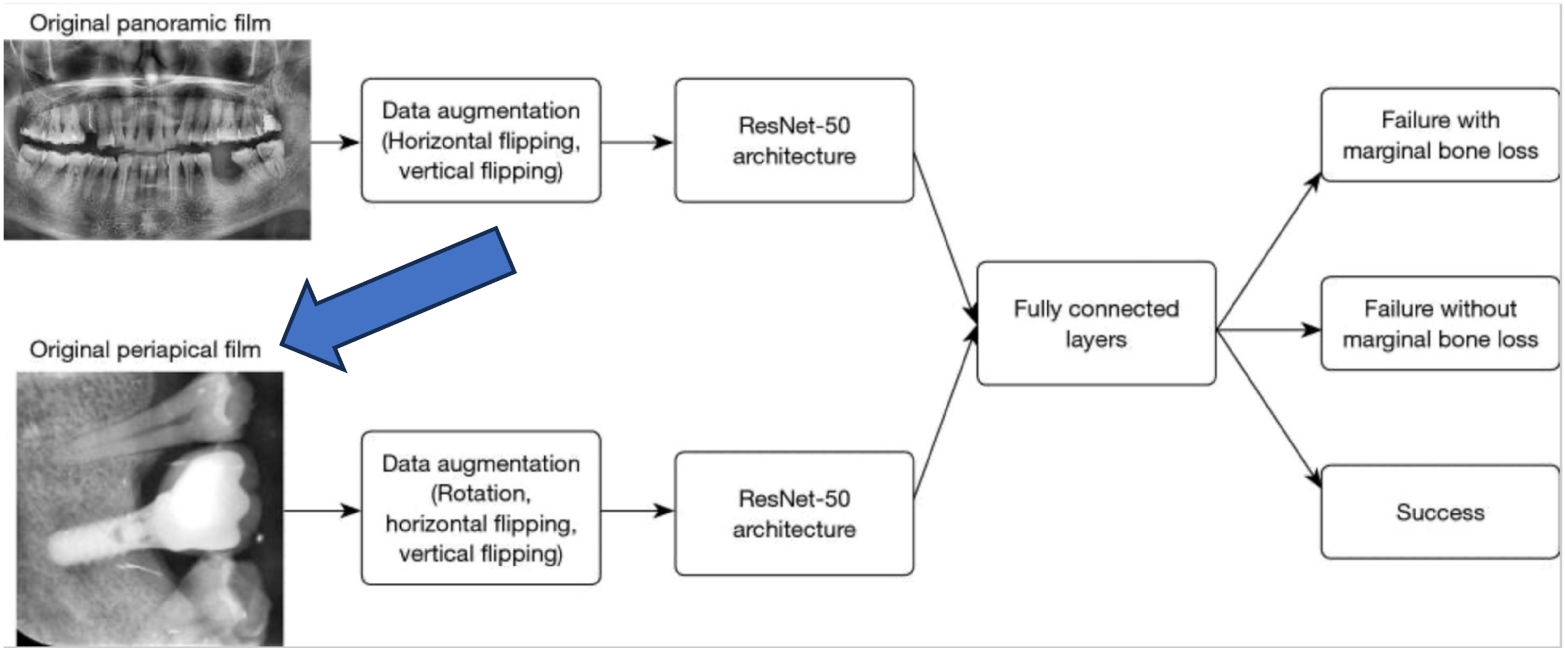
AI and deep learning in implant success. Adapted with permission from (55).

Finally, AI can help predict implant success and implant loss using neural networks (54–56). Dental implant failure is characterized by the features of insufficient bone volume, bone loss, and unfavorable bone quality (57). Zhang et al. (55) developed an effective implant success prediction model by studying the peri-implant alveolar bone pattern on dental periapical and panoramic films (Figure 11). They obtained features using a deep convolutional NN and built a hybrid model to combine panoramic and periapical images. Both the periapical and panoramic images showed a prediction accuracy of 87%.

**Figure 12:**
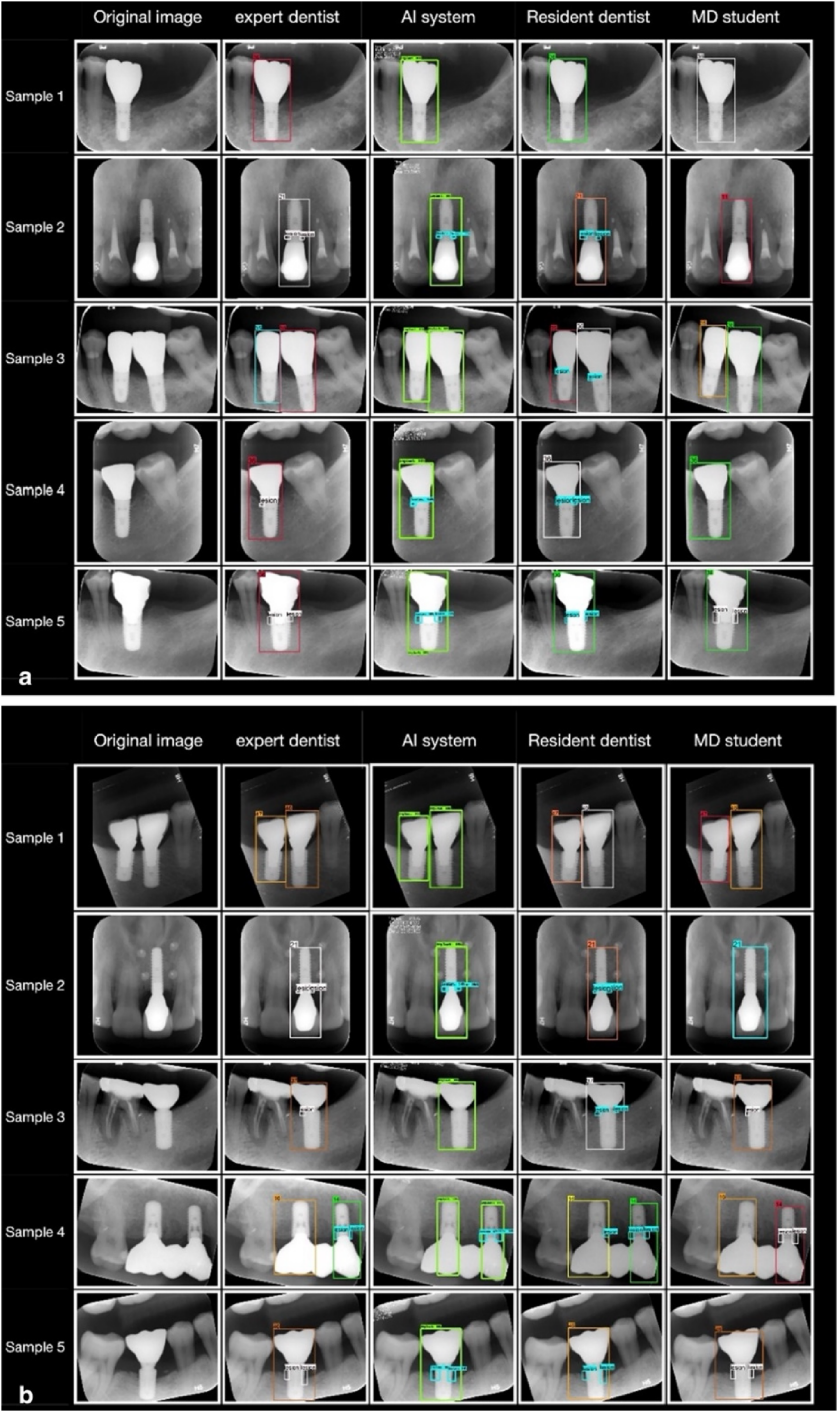
Periapical radiographs showing areas of bone loss detected by neural networks in platform-matched implants **(A)** and platform-switched implants **(B)**. Adapted with permission from (58).

Similarly, Liu et al. (58) studied the accuracy of an AI application for the detection of peri-implant marginal bone loss on periapical radiographs (Figure 12). For this, a Faster region-based convolutional NN (R-CNN) was trained. They found that the evaluation metrics of the AI system were equal to resident dentist. Hence, R-CNN analysis of periapical radiographs is a promising auxiliary diagnostic tool for peri-implant bone loss detection.

### 3.3 AI in orthodontics and pediatric dentistry

The orthodontic diagnosis relies on various analyses such as dental analysis, cephalometric analysis, facial analysis, skeletal analysis, and upper-airway assessment to evaluate the patient's overall profile including facial profile and dental and skeletal relationship (59). Clinical orthodontics requires a significant time for the analysis of each case. With the advancement of computing capabilities and AI algorithms, AI applications are expanding in orthodontics and pediatric dentistry. At present, AI can help in the analysis, diagnosis, and treatment planning, and significantly enhance clinical practice (59, 60). Various AI-driven software such as 3Shape Dental System, Mastro 3D, Uceph, etc. are used widely in orthodontics. The software is undergoing continuous upgrades and developments.

Currently, there is no standardized formula to do extraction for orthodontic alignment and the decision depends on the orthodontists' experience (61). Incorrect decisions can cause irreversible problems such as difficulty closing the extraction space, improper occlusion, and unfavorable profile. AI can assist orthodontists in making decisions and reducing incorrect extractions.

**Figure 13:**
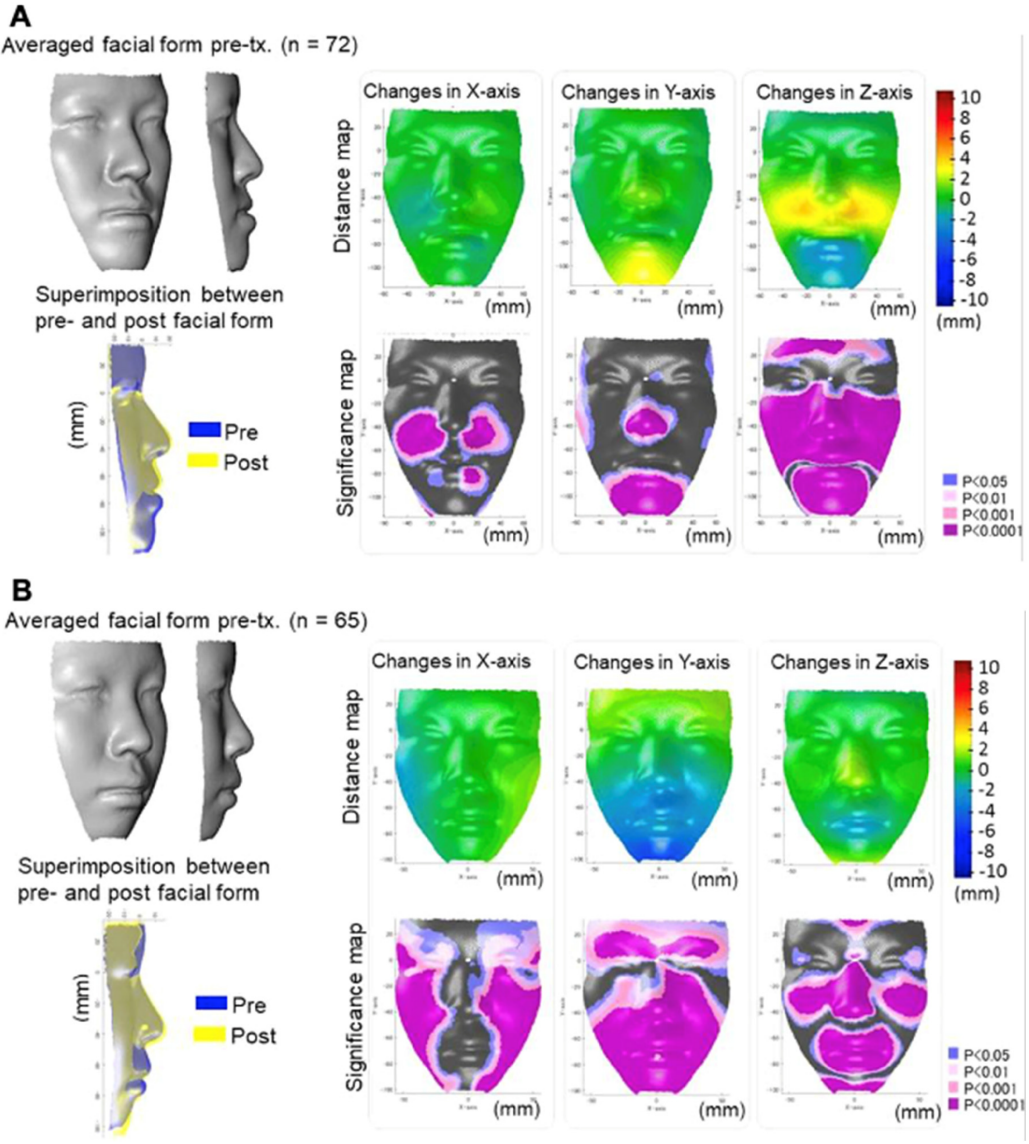
Use of AI in surgical planning in maxillo-mandibular skeletal deformity. **(A)** The actual facial changes in the surgery group for pre-treatment, superimposition, and post-treatment. **(B)** The average actual facial changes in the extraction group for pre-treatment, and the superimposition post-treatment. Adapted with permission from Ref. (63).

Predicting treatment outcomes in orthodontics and pediatric dentistry is important. Currently, AI helps in predicting dental, skeletal, and facial changes thereby guiding treatment planning (62–65). Furthermore, AI to predict skeletal and facial changes after orthodontic treatments. For patients with severe dentofacial deformities, combined orthodontic and orthognathic surgical treatment is required to correct skeletal deformity (66, 67). Lateral cephalograms are being used to assess sagittal skeletal deformities and there is high accuracy in predicting orthognathic surgery diagnosis (68). Tanikawa and Yamashiro (63) developed AI systems that predict the 3D facial profile and morphology after orthognathic surgery and orthodontic treatment based on the results of previous treatment (Figure 13). The success rates of error of <1 mm were 54% and 98% for systems of orthognathic surgery and orthodontic treatment, respectively. The total success rate of error of <2 mm was 100%. Hence, AI systems to predict facial profile and morphology following treatment were clinically acceptable.

### 3.4 AI in prosthetic materials design and fabrication

**Figure 14:**
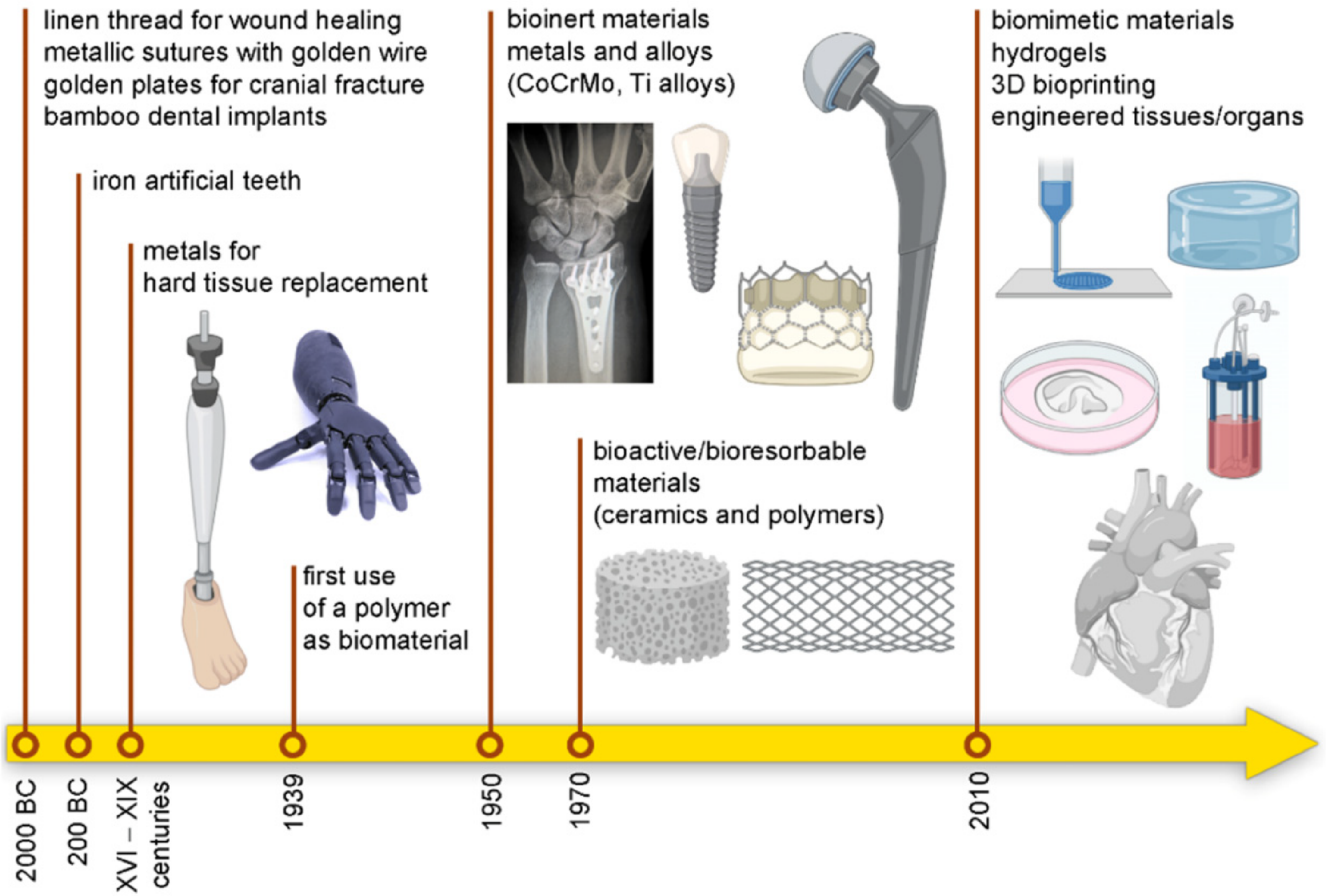
The history and shift of biomaterials: from replacement to regeneration. Adapted with permission from (71).

Over recent decades, a novel discipline within materials science has emerged, focusing on biomaterials. Biomaterials are substances, whether synthetic or natural in origin, that are used to enhance, treat, substitute, or regenerate tissues (69). Currently, biomaterials have extensive applications in medicine and dentistry (70). The trend is shifting from replacement to regeneration (Figure 14) (71). Biomaterials can be broadly classified into metals, polymers, ceramics (including carbon-based materials, ceramics, and glass), and composites (72). Ceramic materials include carbon-based materials, ceramics, and glasses. In the last half-decade, there has been increasing interest in biomaterials for dental applications, and numerous new biomaterials have been introduced for these applications (73–75).

With the wide applications of materials ranging from information, transportation, construction, and biomedicine (3), AI has been implemented in materials science. Recently, AI has made important improvements in rational design and has accelerated the discovery of different biomaterials (3).

**Figure 15:**
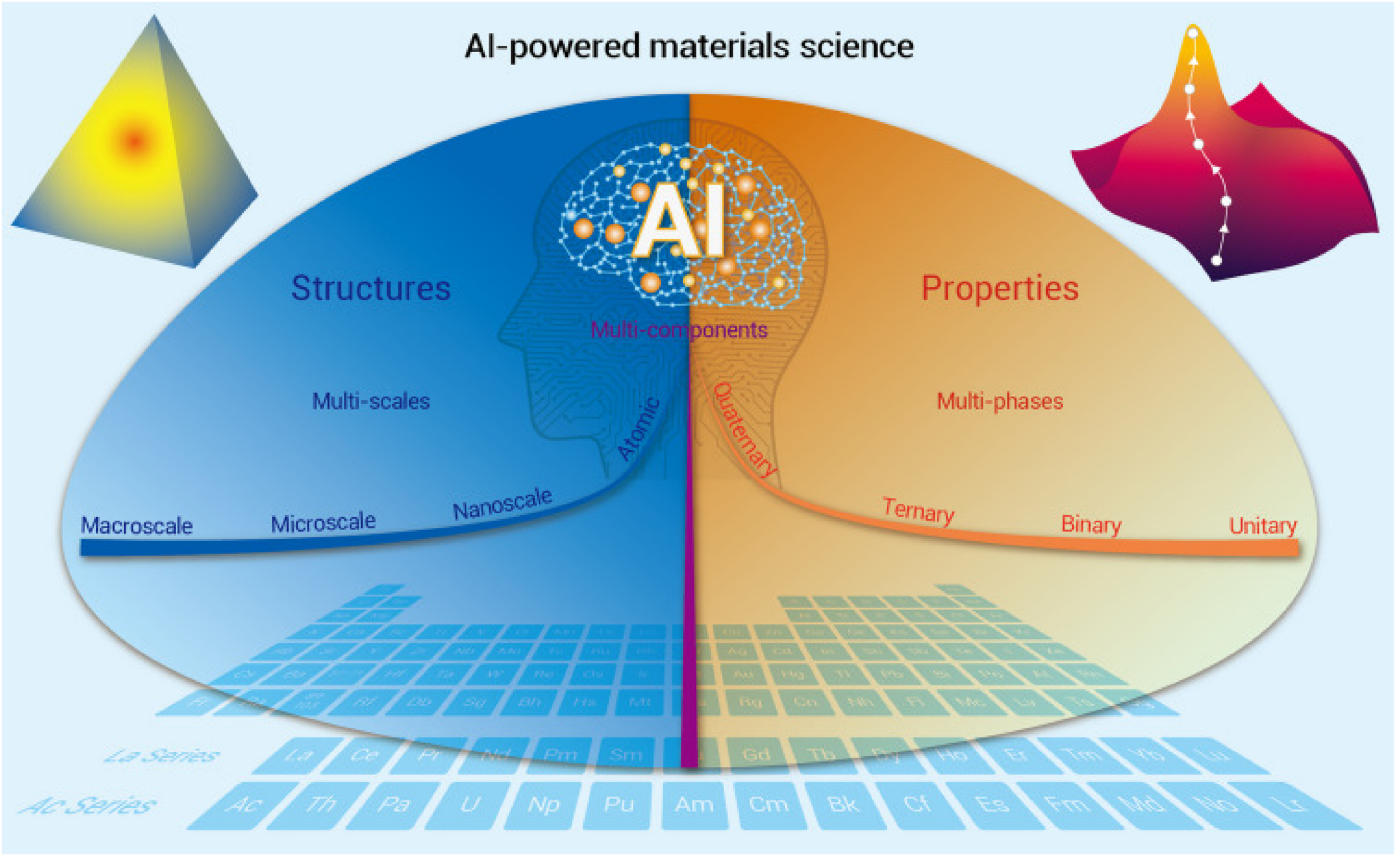
AI-powered development in materials science. Adapted with permission from (3).

With the rapid progress of data handling and algorithms, ML and DL are applied in the search for new biomaterials before actually producing them (76). By incorporating various biomaterial data (such as the integral element, atomic weight and radius, lattice symmetry, magnetism, binding energy, polarization, electronegativity, band energy, and structural property), the machines are trained to improve the design of new materials and predict properties cost-effectively (Figure 15) (3). Data-driven computing and algorithms can help in the simulation and ML of property prediction and material discovery.

AI tools can be adapted for experimental testing of materials and use computing, automation, and ML to calculate material properties such as bulk, interface, and defects (77). In the future, AI techniques can be used to design metals, superconductors, glasses, alloys, magnetocaloric materials, thermoelectric materials, polymeric materials, composite materials, 2D materials, and topological (electronic and phonon) materials (3). A precise AI-based model needs to be developed to forecast the formation energy of a compound to achieve good predictive ability (78).

AI has helped to replace the costly trial and error by developing novel biomaterials. The use of AI methods, notably high-throughput experimentation, has significantly improved the design and production of biomaterials. The major reason for this is the increase in the scope of outcomes to encompass the FDA-endorsed excipient database. The implementation of AI techniques has demonstrated the potential to revolutionize biomaterial development by improving the efficiency and accuracy of research (79). To improve the functionality and application effectiveness of hydrogels, the use of AI has brought about unprecedented advancements in the fields of material design and the optimization of the preparation process. AI has been instrumental in enabling detailed material screening and analysis, performance monitoring, and control. Material characterization analysis has also significantly benefitted from the application of AI technology (80).

AI using 3D printing techniques has been extremely valuable for transforming various data and images to produce prostheses and devices (81). Data collected from 3D instruments from various sources are computed using dental CAD models designed using software, and prostheses are printed using 3D printing technology (82, 83). AI-supported 3D printing facilitates the manufacture of different types of implants and prostheses (3, 84). The prostheses reproduce the normal anatomy precisely and can be customized to help improve function and esthetics. AI can accelerate the fabrication process; hence, the reconstruction process is also quicker (85). Apart from printing the maxillofacial anatomical structures, limbs (i.e., hands and legs) can also be printed (86, 87). The printed limbs can also be supported with myoelectric stimulation support (88, 89). Recent studies have also tried to print bionic ears in models simulating artificial environments, but clinical trials are yet to be conducted soon (90). One advantage of AI is the fabrication of prostheses in children. In children, prostheses need to be changed frequently, which is time-consuming and costly. However, with 3D printing technology using light printing materials, the time and cost can be significantly reduced, and prostheses can be used when needed (3). 3D-printed prostheses in children have better functionality and movement (91).

## 4 Limitations

Although AI holds great promise, it also faces several challenges and ethical considerations. AI in dentistry is rapidly exploring new uses of AI for electronic health records, image analysis, and prosthesis design (92). Atlas-based and statistical shape models are important in capturing the shape, appearance, and location of organs but still a challenge to capture inter-subject variability (8).

Despite a progressive improvement, automatic cephalometry cannot completely replace manual tracing (93) and AI has exceeded the recommended magnitude of error for most cephalometric landmarks (94). It was also found that the AI automatic landmarking on 3D CBCT is found to be less accurate compared to 2D x-rays. Hence, AI-driven radiographic landmarking tracing requires the final supervision and approval of an experienced orthodontist.

Furthermore, important considerations include instilling knowledge of the basic knowledge and application of AI, examining current and potential ethical practices, and discussing its limitations (92, 95). Dental professionals need to understand and acclimatize themselves to such advances for better oral healthcare delivery (96). Finally, AI requires careful governance similar to the governance of physician conduct. Regulatory guidelines are needed regarding safely implementing and assessing AI technology (97).

## 5 Future perspectives

In dentistry, AI technologies can be used as an important tool for treatment planning, diagnosis, prediction of treatment outcomes, and patient-centered care. The integration and continuous improvement of AI has brought significant advancements in dentistry. Various AI-driven software are going through continuous upgrades and developments. The application of AI in dentistry has made promising progress and has great potential for wider clinical applications in the near future.

The future of AI in dentistry extends into dental education, where AI-powered tools offer interactive learning, virtual patient simulations, and personalized feedback (98). Such technologies can allow dental students to practice clinical skills in a controlled, digital environment, preparing them for real-world scenarios.

Although AI has wide applications in dentistry, dental procedures performed by machines without human interaction are not representative of clinical care (25). There is also a risk of fully applying AI in dentistry without human control. AI has shown various errors in analysis and diagnosis. Hence, to avoid such errors, the final evaluation should be done by an experienced dentist.

Human-to-human communication is very difficult to translate directly into computer language and coding (4). Finally, the computational complexity of generating and selecting an atlas is a big challenge. Data safety, security, and privacy are critical aspects of integrating AI into health care (36, 92). Further long-term studies are needed into the specific capabilities to fully integrate AI into clinical practice.

## 6 Conclusion

The application of AI-based systems in dentistry and dental biomaterials is continually increasing. Recent advancement in AI in medical and dental sciences includes multimodal deep learning fusion, speech data detection, and neuromorphic computing. In the future, with the help of generative AI, dentists can prepare patient-specific prostheses for specific oral conditions. The application of AI-based technologies in prosthodontics is desirable in various aspects from clinicians' and patients' points of view. In prosthodontics, they have a tangible influence on widening opportunities for clinicians as well as patients and can be used as an additional simple tool for assembling, handling, and establishing patient-related datasets to deliver individual, patient-centered, and personalized treatment. In orthodontics, AI has contributed to diagnosis, treatment planning, and clinical practice. AI has made important improvements in rational design and has accelerated the discovery of various biomaterials used in dentistry. At present, AI still cannot fully replace human experts and it can serve as an important component in clinical dentistry.
